# Isolation and distribution of rabbit keratocyte precursors

**Published:** 2008-01-30

**Authors:** Tatsuya Mimura, Shiro Amano, Seiichi Yokoo, Saiko Uchida, Tomohiko Usui, Satoru Yamagami

**Affiliations:** 1 Department of Ophthalmology and; 2 Department of Corneal Tissue Regeneration, University of Tokyo Graduate School of Medicine, Bunkyo-ku, Tokyo, Japan

## Abstract

**Purpose:**

To isolate multipotent precursors from the rabbit corneal stroma and to compare the distribution and proliferative capacity of keratocyte precursors obtained from the central and peripheral regions of the corneal stroma.

**Methods:**

The rabbit corneal stroma was divided into a peripheral region (6.0–10.0 mm in diameter) and a central region (6.0 mm in diameter). A sphere-forming assay was then performed to isolate precursors from the stroma of each region. To promote differentiation, isolated sphere colonies were plated in wells with a medium containing fetal bovine serum. Expression of various markers by the sphere colonies and their progeny was examined using immunocytochemistry and/or reverse-transcription polymerase chain reaction (RT–PCR).

**Results:**

The rate of primary sphere formation by cells from the peripheral stroma (51.4±10.1/10,000 cells) was significantly higher than by cells from the central stroma (35.9±3.0/10,000 cells; p=0.00021). Secondary sphere formation rate was significantly higher in the peripheral stroma (45.6±6.4/10,000 cells) than in the central stroma (33.4±2.1/10,000 cells; p=0.00002). Cells from the spheres were positive for CD34 and nestin. Their progeny showed a keratocyte-like spindle shape and expressed vimentin, α-smooth muscle actin, and two neural differentiation markers (microtubule-associated protein-2 and neuron-specific enolase). Expression of nestin and vimentin was confirmed by RT–PCR.

**Conclusions:**

Our findings demonstrate that both the peripheral and central regions of the corneal stroma contain a significant number of precursors, but the peripheral stroma has more precursors with a stronger proliferative capacity than that of cells from the central stroma.

## Introduction

Stem cells or progenitor cells are defined by their capacity for self-renewal and the ability to generate different types of cells (multipotentiality), which leads to the formation of mature tissues, whereas precursor cells are unipotent cells with limited proliferative activity. Regenerative stem cells or precursors can be detected by the sphere-forming assay in various adult tissues, including the central nervous system [1], bone marrow [2], skin [3,4], retina [5], corneal stroma [6-8], and corneal endothelium [7-13].

Corneal stromal cells (keratocytes) as well as corneal endothelial cells are derived from the neural crest [14-16]. Since healing occurs by the proliferation and migration of residual keratocytes from the peripheral part of the stroma [17], keratocyte precursors may have a role in the healing of corneal stromal wounds. Keratocyte precursors may have sufficient self-renewal potential to supply the large number of cells required for wound healing. Corneal stromal wound healing can be expected to be influenced by the location and severity of the corneal injury, but the availability of a pool of viable precursor cells and their proliferative activity could be important determinants of the outcome. Thus, the distribution of keratocyte precursors and their capacity to proliferate has the potential to play an important role in treating corneal stromal diseases, but very little information is available about the distribution and self-renewal capacity of keratocyte precursors in living corneal stroma.

We have used primary sphere formation to isolate keratocyte precursors from the corneas of older donors, which were shipped from the United States two weeks after enucleation. However, no secondary colonies were generated because the proliferative capacity of these cells was not as high as that of younger and fresher donor cells [6]. In the present study, we used rabbit corneas to examine the distribution of corneal keratocyte precursors at a younger age. We isolated precursors with the propensity to develop into corneal keratocyte-like cells from the stroma of rabbit corneas and investigated the distribution and proliferative capacity of precursor cells derived from the central and peripheral regions of the cornea by the sphere-forming assay.

## Methods

### Primary sphere-forming assay

**Figure 1:**
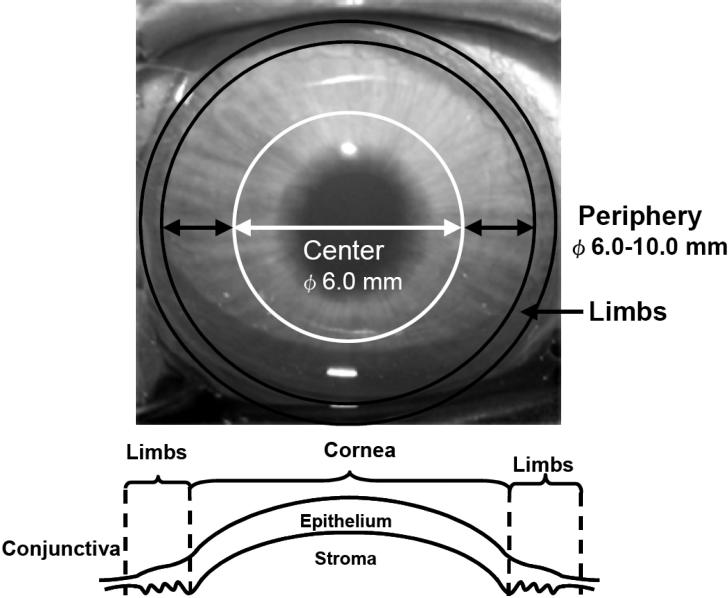
Anterior view of a rabbit cornea and a diagram of the corneal epithelium and stroma. The epithelium was removed from the rabbit corneal stroma by scraping the outer surface of the cornea while the corneal endothelium and Descemet’s membrane were peeled away with fine forceps. To compare the distribution and proliferative capacity of keratocyte precursors obtained from the central and peripheral regions, stromal keratocytes were isolated from tissue specimens obtained from both the peripheral (6.0-10.0 mm in diameter) and central regions (6.0 mm in diameter) using trephines and forceps.

Rabbits were handled in accordance with the ARVO Statement on the Use of Animals in Ophthalmic and Vision Research. Twelve-week-old male New Zealand white rabbits (weighing 2.0-2.4 kg) were obtained from Saitama Experimental Animals Inc. (Saitama, Japan). The animals were anesthetized with intramuscular injections of ketamine hydrochloride (60 mg/kg; Sankyo, Tokyo, Japan) and xylazine (10 mg/kg; Bayer, Leverkusen, Germany) and killed with an overdose of pentobarbital (Nembutal; Dainippon, Osaka, Japan) after which the eyes were enucleated. The eyes were then washed three times with sterile saline and immersed for 5 min in saline containing 10% povidone-iodine (Meiji, Tokyo, Japan) and 50 mg/ml gentamicin (Sigma-Aldrich, St Louis, MO). After further rinsing with saline, the cornea was excised from each eye along the scleral rim. Then, the epithelium was carefully removed from the corneal stroma by scraping the outer surface of the cornea while the corneal endothelium and Descemet’s membrane were peeled away in a sheet from the periphery to the center of the inner surface of the cornea with fine forceps. Samples of corneal stroma were excised from the periphery of the cornea (6.0-10.0 mm in diameter) and from the central region (6.0 mm in diameter) using appropriate trephines (Biopsy Punch, Kai Medical, Gifu, Japan) and forceps as shown Figure 1. These stromal samples were cut into small pieces approximately 1.0 mm in diameter, which were incubated overnight at 37 °C in a basal medium, containing 0.02% collagenase (Sigma-Aldrich). Subsequently, the tissue pieces were washed with phosphate-buffered saline (PBS), incubated in PBS containing 0.2% EDTA for 5 min at 37 °C, and dissociated into single cells by trituration with a fire-polished Pasteur pipette. After centrifugation at 800x g for 5 min, the cells were resuspended in basal medium. The basal medium was Dulbecco’s modified Eagle’s medium (DMEM)/F12 medium supplemented with B27 (Invitrogen, Carlsbad, CA), 20 ng/ml epidermal growth factor (EGF; Sigma-Aldrich), and 40 ng/ml basic fibroblast growth factor (bFGF; Sigma-Aldrich). Isolated keratocytes were counted with a hemocytometer. Viability of the isolated cells was greater than 90% as shown by trypan blue staining (Wako Pure Chemical Industries, Osaka, Japan). The sphere-forming assay was employed for primary culture of the cells [18]. The basal medium, containing a methylcellulose gel matrix (0.8%; Wako Pure Chemical Industries), was used to prevent reaggregation of the cells as described previously [14,19]. Plating was done at a density of 10 viable cells/μl (50,000 cells/well or 2,500 cells/cm^2^) in the uncoated wells of 60 mm culture dishes. To measure the diameter of sphere colonies, culture dishes were observed under an inverted phase-contrast microscope (ELWD 0.3; Nikon, Tokyo, Japan) with a 10× objective lens, and images were analyzed using the NIH image program developed at the United States National Institutes of Health (n=10). The number of spheres per 10,000 cells was calculated for each well. To distinguish growing spheres from dying cell clusters, only those with a diameter of more than 50 μm were counted.

### Secondary sphere formation from primary spheres

For passaging, primary spheres (day 7) were treated with 0.5% EDTA and were dissociated into single cells that were plated into the wells of 60 mm culture dishes at a density of 10 cells/μl. The culture was continued for seven days in a basal medium containing methylcellulose gel matrix to prevent reaggregation. Experiments were performed twice and representative results are shown (n=10).

### Differentiation of sphere colonies

Individual primary spheres (day 7) were transferred to 13-mm glass cover slips coated with 50 μg/ml poly-L-lysine (PLL; Sigma-Aldrich, Tokyo, Japan) and 10 μg/ml fibronectin (BD Biosciences, Billerica, MA) in separate wells as described elsewhere [18]. To promote differentiation, 1% FBS was added to the basal medium, and the culture was continued for another seven days.

**Figure 2:**
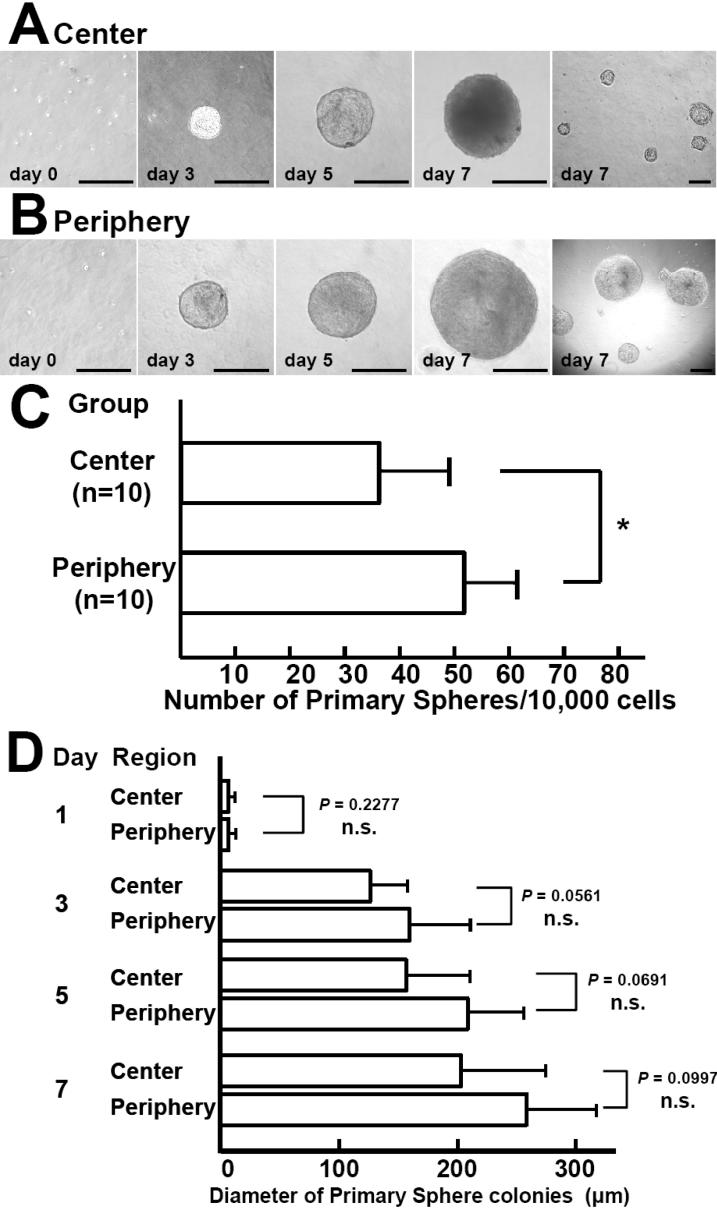
Primary sphere formation by keratocytes from the peripheral and central regions of the rabbit cornea. (**A**, **B**) Stromal cells from the peripheral or central cornea form spheres. Stromal tissue was disaggregated into single cells, which were plated at a density of 10 viable cells/μl in a basal medium containing methylcellulose gel matrix to prevent reaggregation. More than 99% of the cells were single cells on day 0. Growth of a representative sphere is shown until day 7. Scale bar=200 μm. (**C**) The number of primary spheres derived from stromal tissue was compared between the periphery and center of the cornea. The number of sphere colonies obtained from samples of the peripheral stroma (n=10) was significantly higher than that for samples of the central stroma (n=10) after seven days of culture (The asterisk indicates that p=0.00021 and unpaired *t*-test was performed). (**D**) The size of primary sphere colonies derived from samples of peripheral (n=10) and central (n=10) corneal stroma was compared. The mean size of spheres from both regions gradually increased during culture to exceed 250 μm on day 7 (periphery: 258±63 μm versus center: 203±71 μm after seven days, mean±SD). n.s.=not significant.

### Immunocytochemistry

Immunocytochemical examination of the seven-day spheres and their progeny was performed after seven days of adherent culture on the glass cover slips. Cells were fixed with 4% paraformaldehyde (Wako Pure Chemical Industries) in PBS for 10 min. After washing in PBS, the cells were incubated for 30 min with 3% bovine serum albumin (BSA; Sigma-Aldrich) in PBS, containing 0.3% Triton X-100 (BSA/TBST; Rohm & Haas, Philadelphia, PA), to block non-specific binding. Next, the cells were incubated for 2 h at room temperature with the following primary antibodies diluted in BSA/PBST: mouse anti-cytokeratin 3 monoclonal antibody (mAb, AE-5, Progen Biotechnik GMBH, Heidelberg, Germany), mouse anti-\alpha-smooth muscle actin (α-SMA) mAb (1:400; Sigma-Aldrich), mouse anti-vimentin mAb (1:400; Dako, Glostrup, Denmark), mouse anti-nestin mAb (1:400; BD Biosciences), mouse anti-microtubule–associated protein (MAP)-2 mAb (1:400; Chemicon, Temecula, CA), mouse anti-neuron–specific enolase mAb (NSE, 1:400; Dako), mouse anti-CD34 mAb (NCL-END 1:100; Novocastra Laboratories Ltd., Newcastle upon Tyne, UK), and FITC-conjugated mouse anti-5-bromo2’-deoxyuridine (BrdU)/fluorescence mAb (1:100; Roche Diagnostics, Basel, Switzerland). Mouse IgG (1:1000, Sigma-Aldrich) or normal rabbit serum (1:1000, Dako) was used as the control in place of the primary antibody. After being washed in PBS, the cells were reacted for 1 h at room temperature with fluorescence-labeled goat anti-mouse IgG (Alexa Fluor 488, 1:2000; Molecular Probes, Eugene, OR) and fluorescence-labeled goat anti-rabbit IgG (Alexa Fluor 594, 1:400; Molecular Probes) as the secondary antibodies. Finally, fluorescence was detected by observation under a fluorescence microscope (model BH2-RFL-T3 and BX50, Olympus, Tokyo, Japan).

### Extraction of total RNA and reverse-transcription polymerase chain reaction

Total RNA was isolated from keratocytes derived from corneal stroma, primary sphere colonies, and their progeny with a kit (Isogen; Nippon Gene, Tokyo, Japan) according to the manufacturer’s instructions. The isolated RNA was treated with RNase-free DNase I (Stratagene, La Jolla, CA) for 30 min, and cDNA was then obtained with reverse transcriptase (Super Script II; Invitrogen-Gibco, Grand Island, NY). The T12VN primer (25 ng/μl) was used to make the first-strand cDNA. RT–PCR was performed in the absence of reverse transcriptase to act as the negative control. The PCR buffer contained 1.5 mM MgCl_2_ with 0.2 mM of each dNTP (Applied Biosystems, Branchburg, NJ), 0.2 mM of each primer, and 25 unit/l of Amp Taq Gold (Applied Biosystems). After the initial 9 min of denaturing at 95 °C, amplification was performed using a thermal cycler (I-Cycler; Bio Lad Laboratories, Hercules, CA) as follows: 30 cycles in 30 s at 94 °C, 30 s at 60 °C, and 45 s at 72 °C followed by a final 7 min of elongation. The PCR primers were based on the sequences of nestin, ketarin-3, glial fibrillary acidic protein (GFAP), α-SMA, and glyceraldehyde-3-phosphate dehydrogenase (G3PDH). The nestin primers were 5′- TTG AGA C(A/T)C CTG TG(C/A) CAG CCT −3′ (sense) and 5′- CTC TAG AC (T/C) CAC (T/C)GG ATT CT −3′ (antisense); the keratin-3 primers were 5′- GCA GCA GCA GGA TGA GCT G −3′ (sense) and 5′- GTT GAG GGT CTT GAT CTG −3′ (antisense); the keratin-12 primers were 5′- GAG CTG GCC TAC ATG AAG −3′ (sense) and 5′- TTG CTG GAC TGA AGC TGC TC −3′ (antisense); the vimentin primers were 5′- CTT CTC AGC ATC ACG ATG ACC −3′ (sense) and 5′- ATC TAT CTT GCG CTC CTG −3′ (antisense); and the G3PDH primers were 5′- CAT CAC CAT CTT CCA GGA GC −3′ (sense) and 5′- ACA ATG CCG AAG TGG TCG −3′ (antisense). Products were separated by electrophoresis on 1% agarose gel and visualized by staining with ethidium bromide.

**Figure 3:**
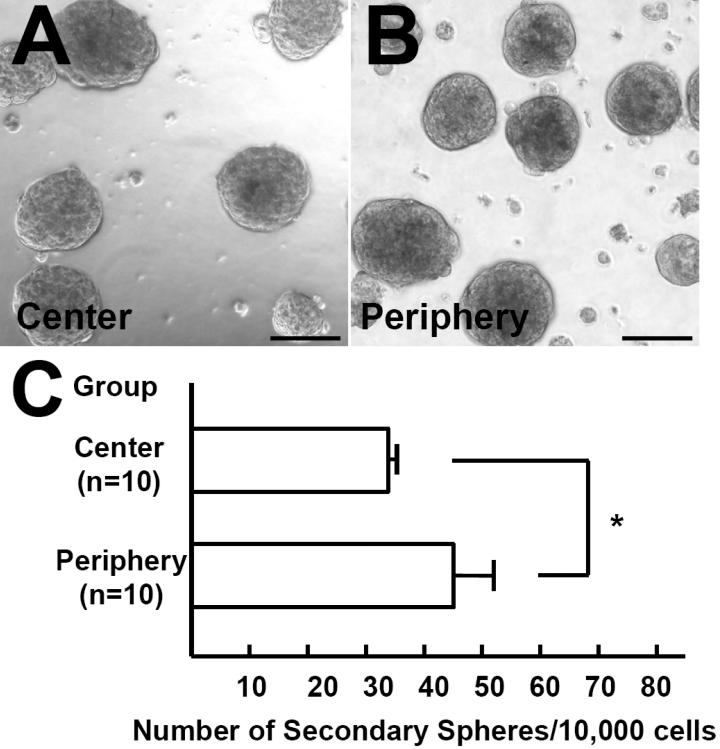
Formation of secondary sphere. (**A**, **B**) Secondary spheres were generated after the dissociation of primary spheres derived from peripheral or central keratocytes. Scale bar=100 μm. (**C**) The re-plating efficiency from primary to secondary colonies was higher for spheres derived from the peripheral stroma than for those from the central stroma (p=0.000025, unpaired *t*-test).

### Statistical analysis

The unpaired *t*-test was used to compare mean values. Significance was defined as p<0.05, and all analyses were performed using a statistical software package (StatView Version 5; Abacus Concepts, Berkeley, CA).

**Figure 4:**
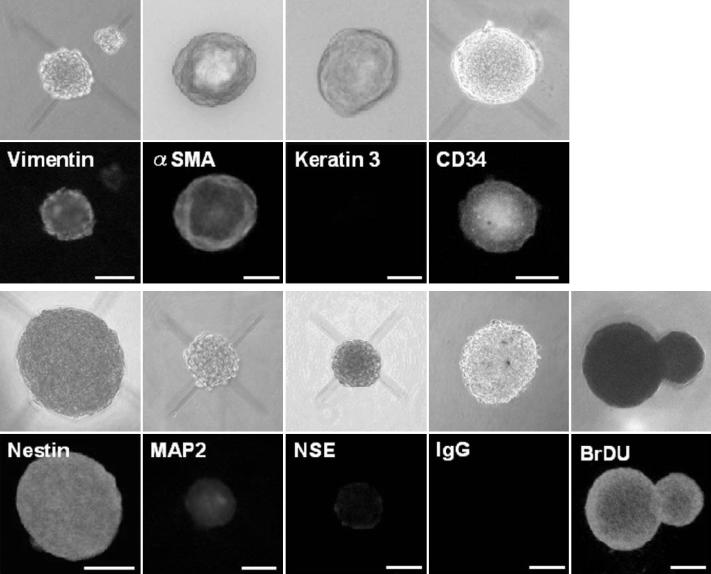
Immunocytochemical analysis of sphere colonies from the peripheral stroma on day 7. Bright-field images and immunostaining of spheres are shown. The spheres were stained for vimentin (a mesenchymal cell marker), \alpha-smooth muscle actin (α-SMA, a mesenchymal cell marker), cytokeratin 3 (a differentiated epithelial cell marker), nestin (a neural stem cell marker), microtubule-associated protein 2 (MAP2, a differentiated neural cell marker), neuron-specific enolase (NSE, a differentiated neural cell marker, and CD34 (a stem cell marker). Each colony is also labeled by BrdU. As a negative control, IgG was used instead of the primary antibody. Scale bar=100 μm.

## Results

### Isolation of sphere colonies

When keratocytes were disaggregated into single cells and cultured for seven days, viable spheres grew larger during that period and non-proliferating cells were eliminated. To compare the density of precursors between the peripheral and central regions of the cornea, primary spheres were isolated separately from the peripheral and central stroma. Photographs of representative spheres obtained from the peripheral, and central regions are shown in Figure 2Aand B. Significantly more spheres (51.4±10.1 per 10,000 cells, mean±SD) were obtained from the peripheral corneal stroma compared with the central stroma (35.9±3.0 per 10,000 cells; p=0.00021; unpaired *t*-test; Figure 2C). No significant differences were noted with respect to the size of primary spheres derived from the two regions after culture for three, five, and seven days, suggesting that there was no difference in the proliferative capacity of precursors obtained from each region (Figure 2D).

**Figure 5:**
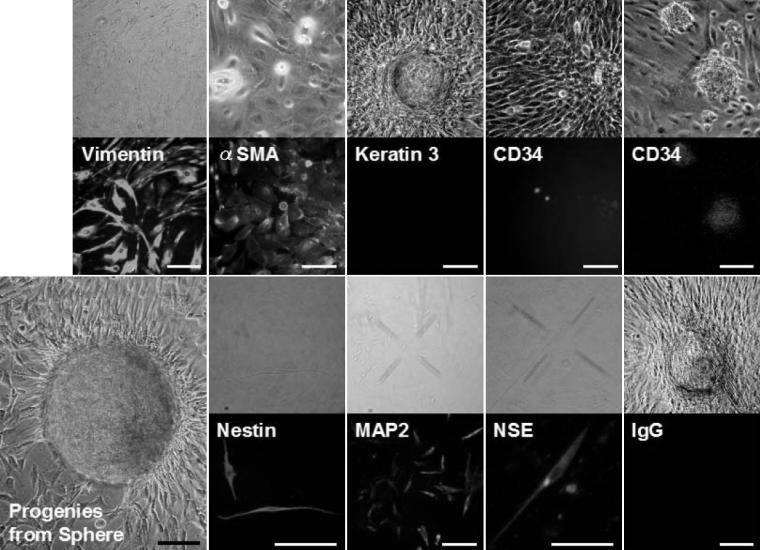
Immunocytochemical analysis of differentiated cells from spheres derived from the peripheral cornea. Cells migrating out from the spheres express α-SMA, MAP2, and NSE, indicating that the colonies contain differentiated mesenchymal and neuronal cells. There is no staining with the control IgG. Scale bar=100 μm.

### Secondary sphere formation

To further evaluate the proliferative capacity of the keratocytes, cells from the primary spheres were passaged under the same culture conditions as those used for the initial growth of the spheres. Secondary spheres were generated after the dissociation of the primary spheres that were derived from the peripheral or central stroma. Photographs of representative secondary spheres are shown in Figure 3A and B. The number of secondary spheres per 10,000 cells was significantly higher when primary spheres that were derived from the peripheral stroma were passaged than spheres from the central stroma (45.6±6.4 versus 33.4±2.1, respectively; p=0.000025; unpaired *t*-test; Figure 3C). Re-plating to generate secondary sphere colonies was less efficient than the generation of primary spheres, indicating that the precursor cells had a limited proliferative capacity.

### Immunocytochemistry and reverse-transcription polymerase chain reaction findings

**Figure 6:**
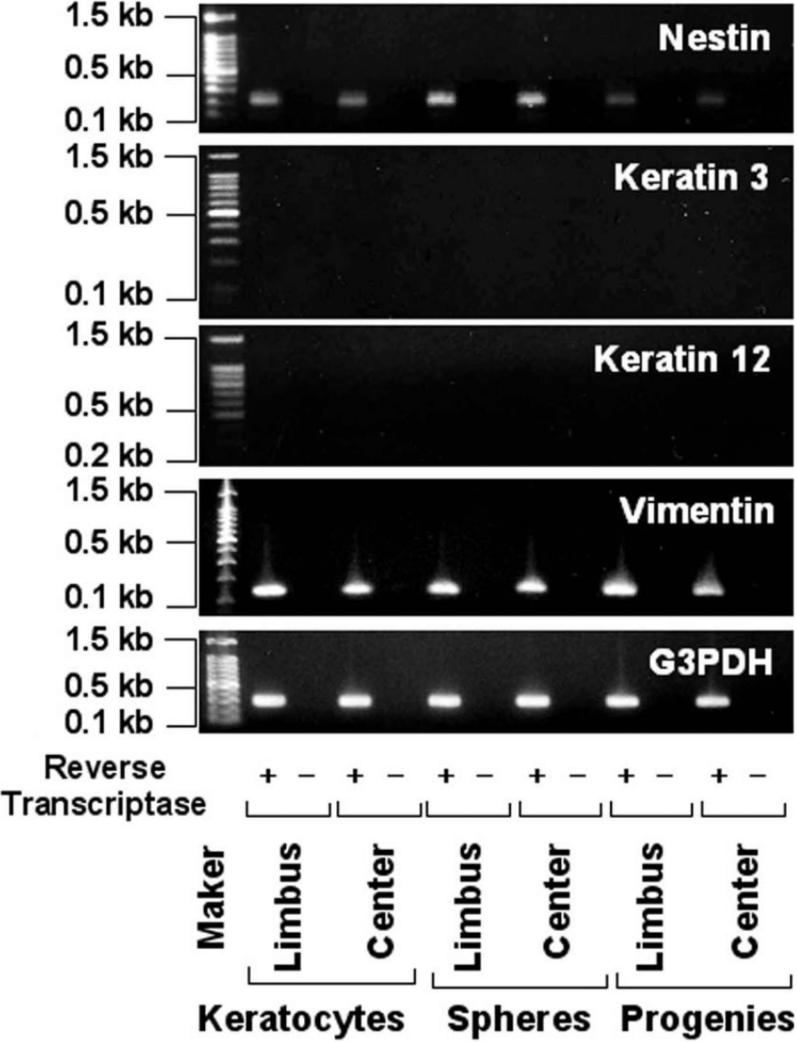
Reverse-transcription polymerase chain reaction analysis of corneal stromal tissue, spheres, and sphere progeny. *G3PDH* gene expression can be detected in all samples except those processed without reverse-transcriptase (RT). Vimentin is expressed by the corneal stromal tissues and the spheres derived from the peripheral or central regions and their progeny but is not detected by PCR of total RNA without RT. Expression of nestin by the progeny is lower than by the spheres from both the peripheral and central regions of the cornea. No expression of keratin 3 or 12 is detected in any of these samples.

Nestin has been used as a marker for the detection of immature neural progenitor cells within multipotential sphere colonies derived from the brain [20], skin [3], inner ear [21], retina [22], corneal stroma [6], and endothelium [9-11]. In addition, the stem cell marker, CD34, was recently suggested to be a useful cell surface marker for human keratocytes [23]. Most cells in the spheres were immunopositive for nestin, CD34, and BrDU (Figure 4). In the normal rabbit’s cornea, CD34 was expressed in the peripheral stroma while a little expression of CD34 was detectable in the central stroma (data not shown). Next, we examined whether the sphere colonies could give rise to cells expressing neural lineage markers. Some cells in the sphere colonies and their progeny expressed microtubule-associated protein 2 (MAP-2; a neural cell marker) and neuron specific enolase (NSE; a marker of neural differentiation; Figure 4 and Figure 5). Most of the cells in the spheres and their progeny were immunoreactive for vimentin (a marker of mesenchymal cells) or αSMA (a marker of fibroblasts) while all were negative for staining by the control, IgG, and the differentiated epithelial cell marker, cytokeratin 3 (Figure 4 and Figure 5). Expression of nestin and vimentin by the spheres and their progeny was confirmed using RT–PCR (Figure 6). Both spheres derived from the peripheral and central regions of the cornea and their progeny displayed the same patterns of immunostaining (data not shown) and mRNA expression (Figure 6).

## Discussion

In this study, we isolated progenitor cells from the rabbit corneal stroma and then investigated the distribution and proliferative capacity of keratocyte precursors derived from the peripheral or central regions of the cornea. As a result, we demonstrated that stroma from both the peripheral and central regions of the cornea contains precursor cells in rabbits, although the peripheral stroma contains significantly more precursors than the central stroma. In addition, secondary sphere formation was significantly more common with cells from the peripheral cornea than with cells from the central region. Despite such differences of their properties, cells derived from keratocyte spheres obtained from the peripheral and central regions of the cornea did not show any differences in the expression of mesenchymal and neural cell markers (data not shown). These findings imply that rabbit keratocyte precursors preferentially reside in the peripheral corneal stroma and have a stronger proliferative capacity compared with cells from the central stroma while precursors from both regions demonstrate similar multipotentiality.

During embryonic development, neural crest cells (from which keratocytes originate) [14,15] show two waves of migration and differentiation related to corneal growth [24,25]. In the first wave, the corneal epithelium forms and synthesizes the primary stroma (such as the periocular mesenchymal cells of neural crest origin) after which neural crest cells migrate to the margin of the optic cup and also migrate between the lens and the corneal epithelium to contribute to the development of the corneal stroma and trabecular meshwork. During the second wave of migration, neural crest cells invade the primary stroma and then undergo differentiation into keratocytes. These embryological processes may be compatible with our finding that more precursors with a high proliferative activity reside near the edge of the corneal stroma, and suggest that these precursors may supply differentiated cells to the central cornea during development.

We evaluated both the proliferative capacity and multilineage potential of keratocyte progenitors and their progeny derived from the peripheral and central corneal stroma. Spheres derived from both the peripheral and central regions of the rabbit cornea showed a high proliferative activity as indicated by BrdU uptake. Their capacity for self-renewal was also demonstrated by the ability of the progeny of individual spheres to form secondary spheres. Moreover, cells in the primary spheres expressed a stem cell marker (CD34) and a neural stem cell marker (nestin) while their progeny expressed mesenchymal markers (vimentin and α-SMA) and neural lineage markers (MAP2 and NSE). These findings indicate that spheres isolated from the corneal stroma of rabbits contained bi-potential precursors and that their progeny displayed the morphologic characteristics of keratocytes. Taken together, these results suggest that precursors from the corneal stroma remain close in nature to the tissue of origin and undergo differentiation into corneal keratocytes. Thus, precursors obtained from the corneal stroma may be more appropriate than multipotential stem cells for tissue regeneration or cell transplantation because such precursors should efficiently differentiate to produce their tissue of origin.

In conclusion, we demonstrated that stroma from the peripheral region of the rabbit cornea contains a higher density of precursors with a stronger proliferative capacity than stroma from the central cornea and that these keratocyte precursors can differentiate into both mesenchymal fibroblasts and neural cells.
